# Adiponectin Induces Oncostatin M Expression in Osteoblasts through the PI3K/Akt Signaling Pathway

**DOI:** 10.3390/ijms17010029

**Published:** 2015-12-25

**Authors:** Chen-Ming Su, Wei-Lin Lee, Chin-Jung Hsu, Ting-Ting Lu, Li-Hong Wang, Guo-Hong Xu, Chih-Hsin Tang

**Affiliations:** 1 Department of Biomedical Sciences Laboratory, Affiliated Dongyang Hospital of Wenzhou Medical University, Dongyang 322100, China; ericsucm@163.com (C.-M.S.); semo1988@163.com (T.-T.L.); 2 School of Pharmacy, China Medical University, Taichung 40402, Taiwan; b1204999@hotmail.com; 3 School of Chinese Medicine, China Medical University, Taichung 40402, Taiwan; jeffrey5991@gmail.com; 4 Department of Orthopedic Surgery, China Medical University Hospital, Taichung 40402, Taiwan; 5 Department of Orthopedics, Dongyang Peoples’ Hospital, Dongyang 322100, China; hustrose666@163.com (L.-H.W.); dyxghd15@163.com (G.-H.X.); 6 Graduate Institute of Basic Medical Science, China Medical University, Taichung 40402, Taiwan; 7 Department of Pharmacology, School of Medicine, China Medical University, Taichung 40402, Taiwan; 8 Department of Biotechnology, College of Health Science, Asia University, Taichung 41354, Taiwan

**Keywords:** rheumatoid arthritis, adiponectin, oncostatin M, osteoblasts

## Abstract

Rheumatoid arthritis (RA), a common autoimmune disorder, is associated with a chronic inflammatory response and unbalanced bone metabolism within the articular microenvironment. Adiponectin, an adipokine secreted by adipocytes, is involved in multiple functions, including lipid metabolism and pro-inflammatory activity. However, the mechanism of adiponectin performance within arthritic inflammation remains unclear. In this study, we observed the effect of adiponectin on the expression of oncostatin M (OSM), a pro-inflammatory cytokine, in human osteoblastic cells. Pretreatment of cells with inhibitors of phosphatidylinositol 3-kinase (PI3K), Akt, and nuclear factor (NF)-κB reduced the adiponectin-induced OSM expression in osteoblasts. Stimulation of the cells with adiponectin increased phosphorylation of PI3K, Akt, and p65. Adiponectin treatment of osteoblasts increased OSM-luciferase activity and p65 binding to NF-κB on the OSM promoter. Our results indicate that adiponectin increased OSM expression via the PI3K, Akt, and NF-κB signaling pathways in osteoblastic cells, suggesting that adiponectin is a novel target for arthritis treatment.

## 1. Introduction

Rheumatoid arthritis (RA) is a chronic autoimmune disorder with unknown etiology. The most typical characterization of RA includes inflammatory synovium and bone destruction because of abnormal immune responses and an accumulation of pro-inflammatory cytokines in the joints [1]. During RA pathogenesis, inflammation results in bone destruction by regulating bone metabolism [2]. Osteoblast-mediated bone formation can repair bone erosion, but the effect of pro-inflammatory cytokines on osteoblast function remains unclear.

Recently, it was shown that in addition to their role in metabolic functions, adipocytes surrounding the RA joints also secrete adipokines that may regulate inflammatory and immune processes [3]. Adiponectin, an adipokine secreted by adipocytes, is associated with metabolic syndromes and pro-inflammatory activity. A previous study demonstrated that the plasma levels of adiponectin were significantly higher in patients with RA than in healthy controls [4]. Adiponectin has not only been proven to play a role in the function of RA synovial fibroblasts, but also to exert different actions in osteoblasts as well. [5]. These include the induction of vascular endothelial growth factor, matrix metalloproteinases, and pro-inflammatory cytokines by osteoblasts [6]. However, the mechanisms accounting for the adiponectin-mediated actions in osteoblasts have not been determined.

Although previous studies revealed a role of osteoclasts in osteoclastogenesis in RA, recent studies have focused on the role of osteoblasts in the process of inflammation and immune response [7]. Oncostatin M (OSM), a pro-inflammatory cytokine, belongs to the interleukin (IL)-6 family [8]. OSM is produced by neutrophils and contributes to inflammation and joint destruction in RA [9]. OSM expression is elevated in the synovial tissues of patients with RA as well as in the subchondral bone in collagen-induced arthritis mouse models [10,11]. In addition, increased OSM expression is regulated by leptin in osteoblasts [12].

In this study, we demonstrated adiponectin-mediated OSM production in osteoblasts. Our results showed that adiponectin up-regulates the expression of OSM through the phosphatidylinositol 3-kinase (PI3K)/Akt/IKK/nuclear factor (NF)-κB signaling pathway in osteoblasts. These results provide an insight into the mechanism of adiponectin function and may have therapeutic value in arthritic pathogenesis.

## 2. Results

### 2.1. Adiponectin Increased OSM Production in Human Osteoblasts

**Figure 1:**
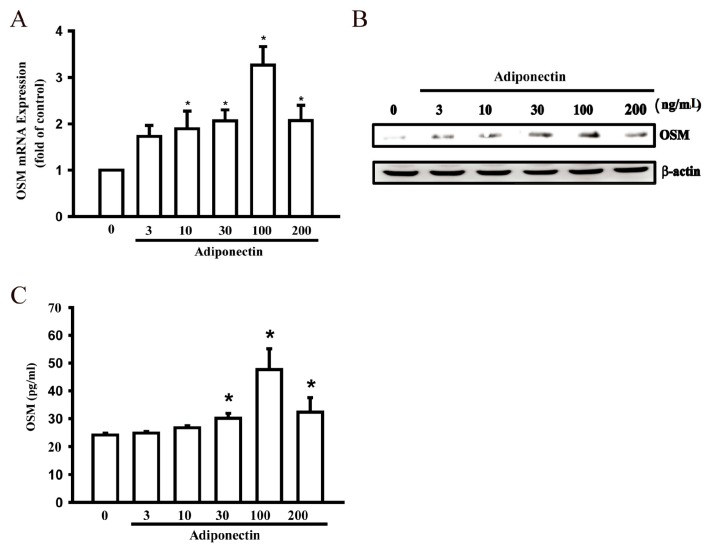
Adiponectin increases oncostatin M (OSM) production in human osteoblasts. (**A**) Osteoblastic cells were incubated with various concentrations of adiponectin (3–200 ng/mL) in OSM mRNA expression and were measured by quantitative polymerase chain reaction (qPCR) (*n* = 6); (**B**) Cells were incubated with various concentrations of adiponectin (3–200 ng/mL) and OSM protein levels were measured by Western blot (*n* = 4); (**C**) Osteoblasts were stimulated with adiponectin (3–200 ng/mL) for 24 h and the supernatant medium was collected and analyzed by enzyme-linked immunosorbent assay (ELISA) (*n* = 5). Results are expressed as mean ± standard error of mean S.E.M. *, *p* < 0.05 compared with control.

Numerous studies have shown that adiponectin promotes the pro-inflammatory response in human macrophages [13,14], indicating a role for adiponectin in RA pathogenesis. In addition, osteoblasts produce inflammatory cytokines that are involved in RA pathogenesis. We used osteoblastic cells to investigate the signaling pathways of adiponectin-mediated OSM production. Treatment of osteoblasts with adiponectin (3–200 ng/mL) for 24 h induced OSM mRNA expression in a concentration-dependent manner (Figure 1A). Adiponectin stimulation resulted in a concentration-dependent rise in OSM protein expression, as highlighted by Western blot analysis and an enzyme-linked immunosorbent assay (Figure 1B,C). These data suggest that adiponectin increased OSM expression.

### 2.2. Signaling Pathways of PI3K Were Involved in Potentiating the Action of Adiponectin

**Figure 2:**
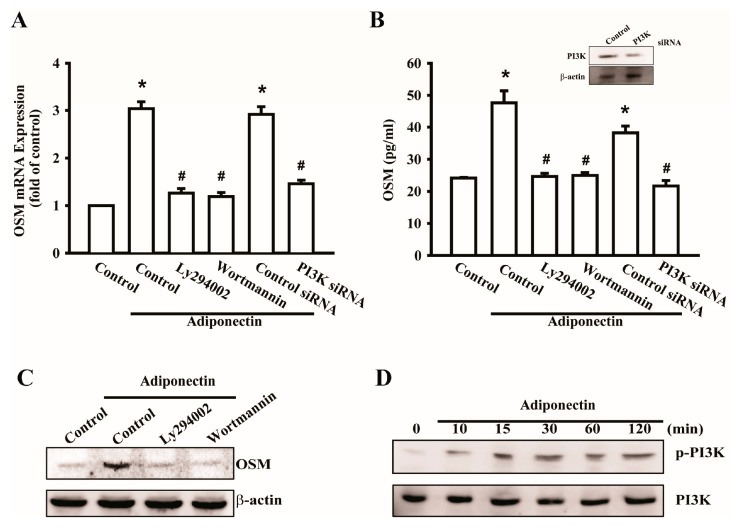
Signaling pathways of phosphatidylinositol 3-kinase (PI3K) involved in potentiating action of adiponectin. (**A**) Osteoblasts were pretreated with PI3K inhibitors, LY294002 (10 μM) or Wortmannin (5 μM), or transfected with p85 short interference RNA (siRNA) (0.5 nM) for 24 h followed by stimulation with adiponectin (100 ng/mL), OSM expression was measured by qPCR (*n* = 6); (**B**) Cells were transfected with p85 siRNA (0.5 nM) for 24 h, the protein level of PI3K was measured by Western blot (**upper-panel**), and supernatant medium was collected to measure OSM expression by ELISAassay (**lower-panel**) (*n* = 4); (**C**) Cells were pretreated with PI3K inhibitors, LY294002 (10 μM) or Wortmannin (5 μM), for 30 min followed by stimulation with adiponectin (100 ng/mL), the protein level of OSM was measured by Western blot (*n* = 5); (**D**) Osteoblasts were incubated with adiponectin (100 ng/mL) in time intervals, and phosphate-PI3K expression was investigated by Western blot (*n* = 6). Results are expressed as mean ± S.E.M. *, *p* < 0.05 compared with control; ^#^, *p* < 0.05 compared with adiponectin-treated group.

A previous study reported that the pro-inflammatory cytokine OSM was associated with the PI3K signaling pathway [15]. We investigated the involvement of PI3K in adiponectin-mediated OSM production. Pretreatment with the PI3K inhibitors Wortmannin and Ly294002 or transfection with p85 siRNA reduced adiponectin-induced OSM mRNA expression (Figure 2A). The supernatant of the culture medium (CM) was collected and analyzed using an OSM enzyme-linked immunosorbent assay kit (Figure 2B). We also determined OSM protein levels by Western blot analysis following pretreatment with PI3K inhibitors to confirm that the PI3K signaling pathway was involved in adiponectin-induced OSM production (Figure 2C). Phosphorylation of p85 was also observed by Western blotting (Figure 2D). These results suggest that adiponectin-mediated OSM expression is regulated through the PI3K signaling pathway.

### 2.3. Involvement of Akt in Adiponectin-Induced OSM Expression in Osteoblasts

**Figure 3:**
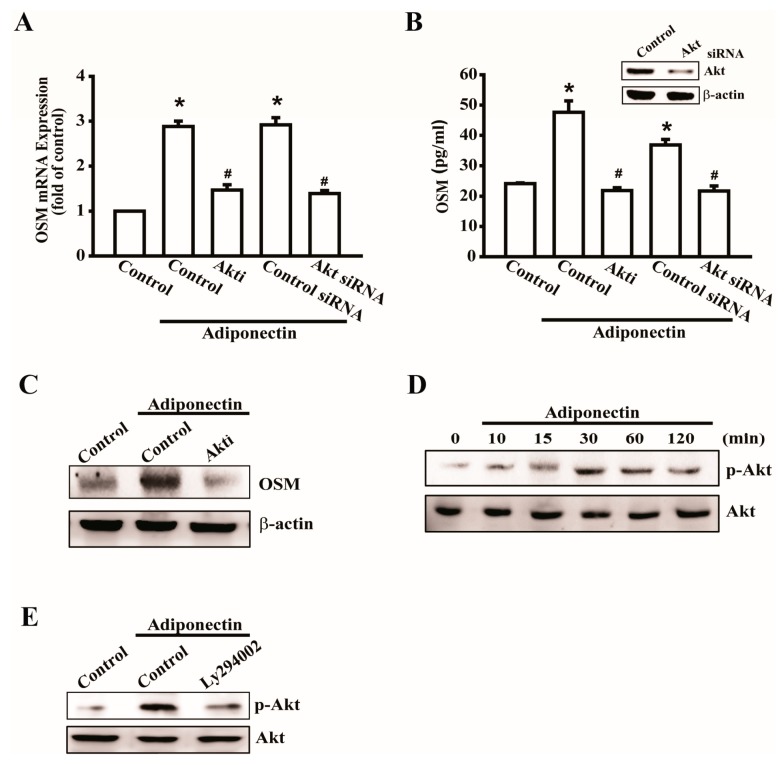
Involvement of Akt in adiponectin-induced OSM expression in osteoblasts. (**A**) Osteoblasts were pretreated with Akt inhibitors (Akti) (20 μM) or transfected with Akt siRNA (0.5 nM) for 24 h followed by stimulation with adiponectin (100 ng/mL), OSM expression was measured by qPCR (*n* = 6); (**B**) Cells were transfected with Akt siRNA (0.5 nM) for 24h, the protein level of Akt was measured by Western blot (**upper-panel**), and supernatant medium was collected to measure OSM expression by ELISA assay (**lower-panel**) (*n* = 5); (**C**) Cells were pretreated with Akti (20 μM) for 30 min followed by stimulation with adiponectin (100 ng/mL), the protein level of OSM was measured by Western blot (*n* = 5); (**D**) Osteoblasts were incubated with adiponectin (100 ng/mL) in time intervals, and phosphate-PI3K expression was investigated by Western blot (*n* = 4); (**E**) Cells were pretreated with PI3K inhibitor, LY294002 (10 μM), for 30 min followed by stimulation with adiponectin (100 ng/mL), phosphate-Akt expression was investigated by Western blot (*n* = 5). Results are expressed as mean ± S.E.M. *, *p* < 0.05 compared with control; ^#^, *p* < 0.05 compared with adiponectin-treated group.

The PI3K-Akt signaling pathway is a common regulator of cellular functions, including protein synthesis, cellular growth, and inflammation [16]. Thus, we evaluated the effect of Akt on adiponectin-induced OSM expression. Pretreatment with an Akt inhibitor or transfection with Akt siRNA decreased adiponectin-induced OSM mRNA expression (Figure 3A). The supernatant of the CM was collected to analyze the expression of secreted OSM (Figure 3B). We further confirmed that Akt is involved in OSM protein expression using Western blotting (Figure 3C); phosphorylated Akt increased in a time-dependent manner in response to adiponectin (Figure 3D). Next, we found that Akt is a downstream signal of PI3K, and pretreatment with a PI3K inhibitor, LY294002, reduced adiponectin-induced Akt phosphorylation (Figure 3E). These results suggest that adiponectin-induced OSM expression was mediated through the PI3K/Akt signaling pathway.

### 2.4. Adiponectin Increased OSM Expression through the NF-κB Pathway

**Figure 4:**
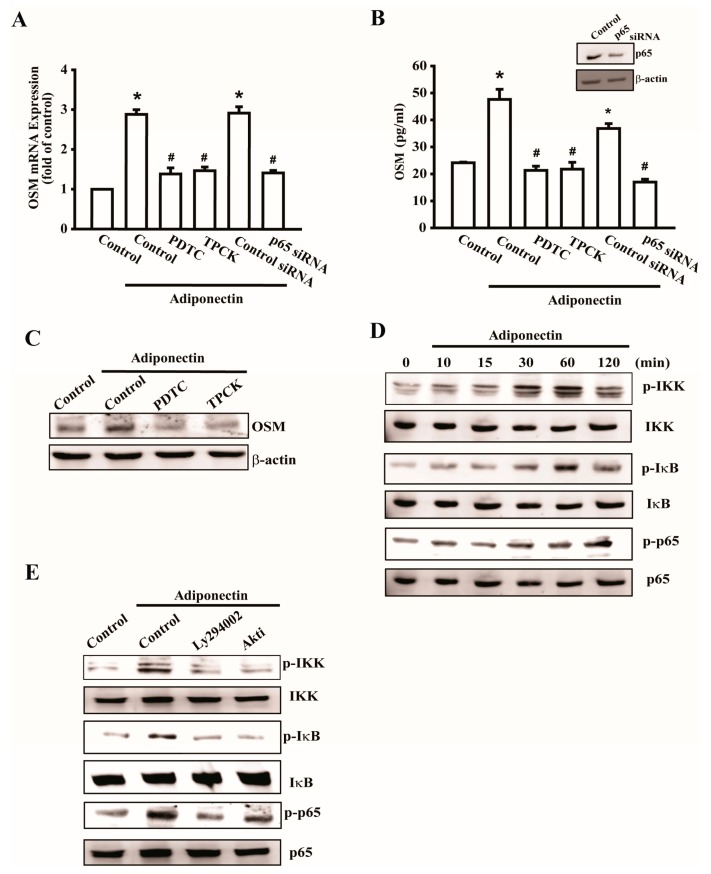
Adiponectin increases OSM expression through nuclear factor-κB (NF-κB) pathway. (**A**) Osteoblasts were pretreated with pyrrolidine dithiocarbamate (PDTC) (10 μM) and N-tosyl-L-phenylalanine chloromethyl ketone (TPCK) (10 μM) or transfected with p65 siRNA (0.5 nM) followed by stimulation with adiponectin (100 ng/mL), the mRNA expression of OSM were analyzed by qPCR (*n* = 5); (**B**) Cells were transfected with p65 siRNA for 24 h, the protein level of p65 was measured by Western blot (upper-panel), and supernatant medium was collected to measure OSM expression by ELISA assay (lower-panel) (*n* = 5); (**C**) Cells were pretreated with PDTC and TPCK for 30 min followed by stimulation with adiponectin (100 ng/mL), the protein level of OSM was measured by Western blot (*n* = 6); (**D**) Cells were incubated with adiponectin in time intervals, and phosphate-IKK, -IκB, and -p65 expression were investigated by Western blot (*n* = 5); (**E**) Cells were pretreated with PI3K inhibitor, LY294002 (10 μM), or Akt inhibitor (20 μM) for 30 min followed by stimulation with adiponectin (100 ng/mL), phosphate-IKK, -IκB, and -p65 expression were investigated by Western blot (*n* = 6). Results are expressed as mean ± standard error of mean S.E.M. *, *p* < 0.05 compared with control; ^#^, *p* < 0.05 compared with adiponectin-treated group.

**Figure 5:**
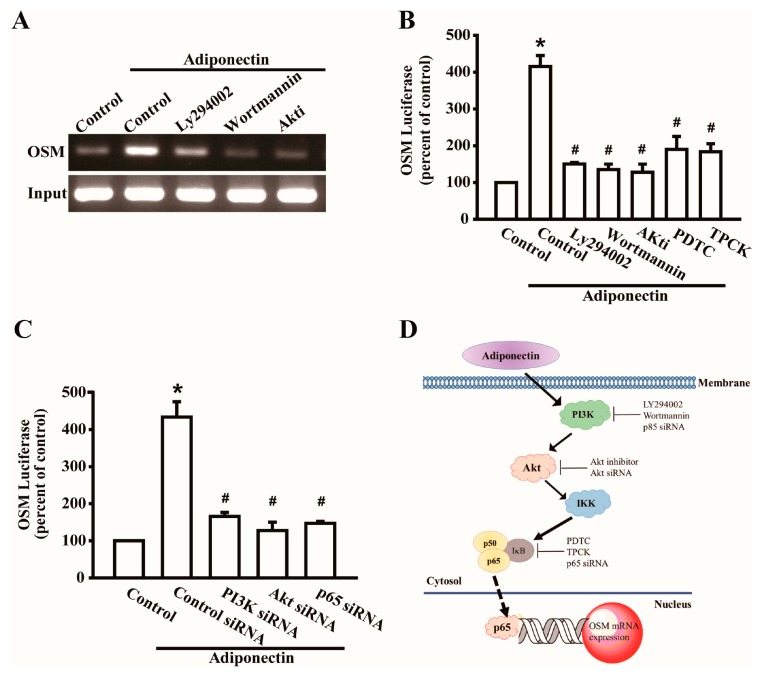
Adiponectin triggers p65 binding to OSM promoter. (**A**) Osteoblasts were pretreated with PI3K inhibitors, LY294002 (10 μM) or Wortmannin (5 μM), or Akt inhibitor (20 μM) for 30 min stimulated with adiponectin (100 ng/mL) for 60 min, and the chromatin immunoprecipitation assay was performed (*n* = 8); (**B**) Osteoblasts were pretreated with LY294002, Wortmannin, Akt inhibitor, PDTC, or TPCK for 30 min followed by treatment with adiponectin (100 ng/mL) (*n* = 6); (**C**) Osteoblasts were transfected with PI3K, Akt, or p65 siRNA (0.5 nM) followed by treatment with adiponectin (100 ng/mL). OSM-luciferase activity was measured, and the results were normalized to the β-galactosidase activity (*n* = 6). Results are expressed as mean ± S.E.M. *, *p* < 0.05 compared with control; ^#^, *p* < 0.05 compared with adiponectin-treated group; (**D**) The schematic diagram of the signaling pathway showed adiponectin-induced OSM expression in osteoblastic cells. Solid arrow means signal transduction and dotted arrow means transcription.

Previous studies have shown that the transcription factor NF-κB is involved in bone resorption by osteoclasts, but is rarely involved in bone metabolism by osteoblasts [17,18]. We further investigated whether NF-κB activation is involved in adiponectin-mediated OSM expression by using the NF-κB inhibitors pyrrolidine dithiocarbamate (PDTC) and N-tosyl-L-phenylalanine chloromethyl ketone (TPCK) or p65 siRNA. Pretreatment with PDTC or TPCK or transfection with p65 siRNA abolished adiponectin-enhanced OSM expression (Figure 4A–C). Additionally, stimulation of osteoblasts with adiponectin increased IKK, IκB, and p65 phosphorylation in a time-dependent manner (Figure 4D). Pretreatment with inhibitors of PI3K or Akt reduced the adiponectin-induced phosphorylation of IKK, IκB, and (Figure 4E). These results highlighted the importance of NF-κB in response to adiponectin-mediated OSM expression. We continued to evaluate p65 binding to the OSM promoter after adiponectin stimulation. Based on the results of a chromatin immunoprecipitation (ChIP) assay, adiponectin-induced binding of p65 at the OSM promoter was attenuated by Ly294002, Wortmannin, and an Akt inhibitor (Figure 5A). In addition, to further verify the upstream signaling pathway involved in adiponectin-induced OSM activation, OSM-luciferase activity was measured. We constructed an OSM promoter-luciferase reporter plasmid to detect OSM activation after adiponectin treatment in osteoblasts. We found that adiponectin stimulation of OSM luciferase activity was decreased by Ly294002, Wortmannin, Akt inhibitor, PDTC, and TPCK (Figure 5B). Co-transfection of cells with siRNA against p85, Akt, and p65 also reduced adiponectin-enhanced OSM luciferase activity (Figure 5C). A diagram of the signaling pathway is shown in Figure 5D. Taken together, these data suggest that activation of the PI3K/Akt/NF-κB pathway is required for adiponectin to increase OSM expression in human osteoblasts.

## 3. Discussion

Although previous studies have demonstrated the impact of inflammation in osteoclasts, an increasing number of recent studies have focused on the role of osteoblasts in RA pathogenesis [11,19]. Accumulating reports have shown that subchondral bone leads to the degeneration of articular cartilage that may be associated with pro-inflammatory cytokines [20,21]. In metabolism syndrome, adiponectin is a beneficial circulating adipokine with pleiotropic functions, including glucose metabolism and lipid elimination. In contrast, adiponectin and its receptors have been reported to be involved in bone metabolism in osteoblasts [22]. Physiological adiponectin concentration in blood is 1–10 μg/mL, and our previous report used adiponectin at the concentration of 3 μg/mL in synovial fibroblasts [23]. In addition, it has been indicated that low concentration of adiponectin (10 ng/mL) promoted chondrosarcoma migration [24]. In this study, we found that osteoblastic cells were treated with adiponectin at the concentration of 100 ng/mL to induce both OSM protein levels and gene expression. Collectively, adiponectin might cause particular effects on different cells. On the other hand, adiponectin three isoforms, low-molecular weight (LMW), medium-molecular weight (MMW), and high-molecular weight (HMW), have differential effects on gene expression [25]. Both LMW and MMW present in cerebrospinal fluid and enhance nitric oxide production, and HMW improves hepatic insulin sensitivity and enhances monocyte release of IL-6 [25]. In this study, the recombinant adiponectin we purchased is from PeproTech, but it does not supply clear information of the composition rate of adiponectin. Therefore, *in vitro* or *in vivo* effects on the distinction of different isoforms of adiponectin are a limitation of this study. Thus, multiple studies have demonstrated the harmful role of adiponectin, which exacerbated inflammation during RA pathogenesis; additionally, the serum and synovial fluid concentrations of adiponectin are higher in patients with RA [26,27]. The specific mechanism by which adiponectin affects the inflammation and immune responses in RA pathogenesis remains unclear.

OSM, a gp130 family member, is associated with IL-6 during bone regulatory activity and inflammation [28,29]. OSM is produced both by several cells derived from hemopoietic stem cells and by the osteoblast lineage in the bone microenvironment, which thereby perform both pro-anabolic action of osteoblasts and potentially catabolic action of osteoclasts [30]. During RA pathogenesis, inflammatory macrophages produce OSM pro-anabolic influences [31]. In addition, our previous studies indicated a critical role for OSM in osteoblasts during RA pathogenesis [11,12,32]. Here, we found an interesting result concerning that adiponectin-induced OSM expression might be associated with its pro-anabolic action of osteoblasts, but the potentially catabolic action of osteoclasts needs to be further evaluated in the future.

Previous studies revealed an important role for adiponectin receptors in some post-receptor signaling mechanisms, including the PI3K/Akt signaling pathway [33]. Our recent study demonstrated that adiponectin promoted angiogenesis in human chondrosarcomas through adiponectin receptors and the PI3K/Akt/mTOR/HIF signaling pathway [34]. In addition, adiponectin induced AMPK/c-Jun/AP-1 signaling pathways in synovial fibroblasts [23]. Here we demonstrated that adiponectin activated OSM expression through the PI3K/Akt/IKK/NF-κB signaling pathway. However, if adiponectin also induced other similar signaling pathways, such as PI3K/Akt/mTOR/HIF or AMPK/c-Jun/AP-1 or if these different signaling pathways interfere each other following adiponectin treatment requires further exploration in the future.

The NF-κB pathway plays a critical role in chronic inflammatory responses and is constitutively activated by PI3K-dependent phosphorylation of IKK [35]. A recent study revealed that OSM production is regulated primarily through NF-κB at the transcription level via the integrin receptor/PI3K/Akt signaling pathways in RA pathogenesis [32]. However, another report indicated that NF-κB transcription and nuclear translocation were unaffected by a neuropeptide in mouse calvarial osteoblasts [18]. Our data showed that stimulation of osteoblasts with adiponectin increased NF-κB translocation and activated p65 biding to NF-κB at the OSM promoter, indicating that NF-κB is one of the most important transcription factor binding sites for adiponectin-induced OSM expression during inflammatory responses. In addition, we also transfected OSM luciferase as an indicator of OSM activity and observed that adiponectin induced an increase in OSM activity that was reduced by the upstream inhibitors Ly294002, Wortmannin, Akt inhibitor, PDTC, and TPCK and siRNA against PI3K, Akt, and p65. Taken together, our results indicated that adiponectin acted through the PI3K, Akt, and NF-κB signaling pathways to induce OSM production in human osteoblasts. In conclusion, we found that adiponectin augmented OSM expression by activating the PI3K/Akt/NF-κB signaling pathways in osteoblasts, suggesting that the connection between adiponectin and pro-inflammatory cytokine OSM could influence osteoblastic function under RA pathogenesis. These results increase our understanding of the mechanisms by which adiponectin induces OSM production underlying inflammatory responses and revealed a potential therapeutic target of arthritis.

## 4. Materials and Methods

### 4.1. Materials

Rabbit polyclonal antibody specific for phosphate-p85, a heterodimer of phosphatidylinositol 3 kinase (PI3K), p-Akt, p-IKK were purchased from Cell Signaling Technology (Danvers, MA, USA). Rabbit polyclonal antibodies specific for PI3K, Akt, IKK, NF-κB, β-actin, and mouse polyclonal antibodies specific for OSM were purchased from Santa Cruz Biotechnology (Santa Cruz, CA, USA). The recombinant human adiponectin was purchased from PeproTech (Rocky Hill, NJ, USA). PI3K inhibitors (Wortmannin and Ly294002), Akt inhibitor, and NF-κB inhibitors pyrrolidine dithiocarbamate (PDTC) and L-1-tosylamido-2-phenylenylethyl chloromethyl ketone (TPCK) were purchased from Sigma-Aldrich (St. Louis, MO, USA). Small-interfering RNAs (siRNAs) against p85, Akt, and p65 were purchased from Dharmacon Research (Lafayette, CO, USA). OSM ELISA kit was purchased from R&D Systems (Minneapolis, MN, USA). DMEM, fetal bovine serum (FBS), and all the other cell culture reagents were purchased from Gibco life technologies (Grand Island, NY, USA).

### 4.2. Cell Culture

The human osteoblast-like cell line MG-63 was purchased from the American Type Culture Collection (Manassas, VA, USA). MG-63 cells were cultured in DMEM supplemented with 10% FBS (Invitrogen, Carlsbad, CA, USA) and antibiotics (100 U/mL penicillin G and 100 mg/mL streptomycin). Cultures were maintained in a humidified atmosphere of 5% CO_2_ at 37 °C.

### 4.3. Western Blot Analysis

Cellular lysates were prepared from previous studies [36]. Proteins were resolved through sodium dodecyl sulfate-polyacrylamide gel electrophoresis and transferred to Immobilon polyvinyl difluoride membranes (Millipore, Billerica, MA, USA). The blots were blocked with 4% non-fat milk for 1 h at room temperature and then probed with rabbit anti-human antibodies against p-PI3K, p-Akt, p-IKK, p-p65, and mouse anti-human antibodies against OSM for 1 h at room temperature. After washing three times, the blots were subsequently incubated with a goat anti-rabbit or goat anti-mouse peroxidase-conjugated secondary antibody for 1 h at room temperature. The blots with horseradish peroxidase-labeled substrate were detected by enhanced chemiluminescence and visualized by using a Fujifilm LAS-3000 chemiluminescence detection system (Fujifilm, Tokyo, Japan). All results are expressed for more than four independent experiments.

### 4.4. Quantitative Real-Time Polymerase Chain Reaction

Total RNA was extracted from osteoblasts using a TRIzol kit (MDBio, Taipei, Taiwan). RNA quality and yield were determined by absorbance at 260-nm measurements performed with a Nanovue Spectrophotometer (GE Healthcare, Madison, WI, USA). Complementary DNA was synthetized from 1 μg total RNA using a Moloney Murine Leukemia Virus Reverse Transcription kit (Invitrogen) following the manufacturer’s recommendations. Quantitative real-time polymerase chain reaction (qRT-PCR) analysis was carried out with SYBR One-Step RT-PCR Master Mix (Applied Biosystems, Foster City, CA, USA). All target gene primers were purchased from Applied Biosystems and the sequences were as follows: GAPDH, forward-AGCCACATCGCTCAGACAC, reverse-GCCCAATACGACCAAATCC; OSM, forward-AGTACCGCGTGCTCCTTG, reverse-CCCTGCAGTGCTCTCTCAGT. qPCR assays were carried out in triplicate using a StepOnePlus sequence detection system (Applied Biosystems), according to the manufacturer’s instructions. All results are expressed for six independent experiments performed in duplicate.

### 4.5. Enzyme-Linked Immunosorbent Assay

Osteoblasts were cultured in 24 well culture plates. Cells were treated with adiponectin and then incubated at 37 °C for 24 h. To examine the downstream signaling pathways responsive to adiponectin treatment, cells were pretreated with various inhibitors for 30 min before the addition of adiponectin (100 ng/mL). After incubation, the supernatant medium was collected and stored at −80 °C until the assay was performed. OSM in the medium was assayed using an OSM enzyme-linked immunosorbent assay (ELISA) kit according to the manufacturer’s instructions. All results are expressed for five independent experiments.

### 4.6. Plasmid Construction

Plasmid pGL2-Basic (Promega, Madison, WI, USA) and primers of the insert were used to generate construct of the OSM promoter as previously described [32]. Briefly, human OSM was designed between restriction enzymes NheI and BglII and amplified by PCR. After digestion, the insert was subcloned into the luciferase reporter vector pGL2-Basic. The construct was confirmed by sequencing using the Applied Biosystems 3730XL DNA Analyzer (Thermo Fisher Scientific Inc., New York, NY, USA).

### 4.7. Transfection and Reporter Gene Assay

Control ON-TARGETplus small interfering RNA (siRNA) and ON-TARGETplus siRNAs against p85, Akt, and p65 were purchased from Dharmacon Research (Lafayette, CO, USA). Transient transfection of siRNA (0.5 nM) was carried out using Dharma FECT 1 transfection reagent (Thermo, Waltham, MA, USA) for 24 h, according to the manufacturer’s instructions. For the reporter assay, cells were co-transfected with OSM luciferase (0.5 μg) and β-galactosidase expression vector (0.5 μg) for 24 h using Lipofectamine 2000 (Invitrogen), according to the manufacturer’s protocol. All results are expressed for four independent experiments.

### 4.8. Chromatin Immunoprecipitation Assay

Chromatin immunoprecipitation (ChIP) analysis was performed as described previously [11]. DNA, immunoprecipitated with an anti–p65 antibody, was purified and extracted with phenol–chloroform and then amplified using PCR. PCR products were resolved by electrophoresis in a 1.5% agarose gel and visualized under ultraviolet light. Primers were utilized to amplify across the OSM promoter region as previously describe [32]. All results are expressed for more than four independent experiments.

### 4.9. Statistical Analysis

Data are presented as mean ± standard error of the mean. Statistical analysis of comparisons between the experimental groups and controls was performed using Student’s *t* test. Statistical comparisons of more than 2 groups were performed using one-way analysis of variance with Bonferroni’s *post-hoc* test. In all cases, *p* < 0.05 was considered significant.
